# Bip inhibition in glioma stem cells promotes radiation-induced immunogenic cell death

**DOI:** 10.1038/s41419-020-03000-z

**Published:** 2020-09-22

**Authors:** Wei Yang, Zenghe Xiu, Yuping He, Wenpeng Huang, Yanyan Li, Ting Sun

**Affiliations:** 1 State Key Laboratory of Radiation Medicine and Protection, School of Radiation Medicine and Protection and Collaborative InnovationCenter of Radiation Medicine of Jiangsu Higher Education Institutions, Soochow University, Suzhou, Jiangsu China; 2 Neurosurgery and Brain and Nerve Research Laboratory, The First Affiliated Hospital of Soochow University, 215006 Suzhou, Jiangsu China

**Keywords:** Cancer stem cells, Immune cell death

## Abstract

Tumor regression in sites distant to the irradiated field are thought to be associated with emission of damage-associated molecular patterns (DAMPs) molecules and generation of immunogenic cell death (ICD). Glioma stem cells (GSCs) are resistant to high doses of radiation, and ultimately select the outgrowth of a more aggressive tumor. This study showed high-dose IR triggered fewer DAMPs molecules exposure and release in GSCs comparing to matched non-GSCs. Downregulation of binding immunoglobulin protein (Bip) promoted IR-mediated endoplasmic reticulum stress to generate DAMPs molecules by PERK and IRE1-α phosphorylation, and increased dendritic cells mature and effector T lymphocytes activation. GSCs treated with Bip knockdown and IR efficiently prevented tumor generation, and reduced post-radiotherapy tumor recurrence. These data suggest that Bip plays a critical role in inhibition of IR-induced ICD in GSCs, and Bip inhibition may be a promising strategy on adjuvant therapy by ameliorating tumor immune microenvironment.

## Introduction

Glioblastoma (GBM) is the most aggressive primary brain tumor with a high mortality rate. Despite advanced multimodality treatment consisting of resection, radiotherapy (RT), chemotherapy, and other adjuvant therapy, median survival remains dismal at 12–15 months^1,2^. GBM patients typically respond initially to therapy, but tumor ultimately relapses within the high-dose irradiation field, suggesting the presence of a subpopulation of resistant cells. The small and rare cell subpopulation, termed glioma stem cells (GSCs), with stem-like properties including self-renewal, multi-lineage differentiation potential and resistance to conventional treatments, has the ability to recapitulate the entire cell repertoire of the whole tumor^3,4^. RT may initially reduce the bulk of the tumor by targeting non-GSCs, however, GSCs can resist even high doses of radiation to ultimately select the outgrowth of a more aggressive tumor^5^. Many, although not all, clinical trials have failed to show a benefit to radiation dose escalation, radiosurgery boost, or brachy therapy boost.

RT is generally used as a primary therapy of localized tumors by inducing DNA damage and blocking the cell division. Increasing evidences reported tumor regression observed following RT alone^6^ or combination with immunotherapy^7,8^ in sites distant to the irradiated field recently. RT provokes the emission of immunogenic signals conveyed by damage-associated molecular patterns (DAMPs) molecules such as plasma membrane-exposed calreticulin (CRT), ATP and high mobility group box1 (HMGB1) during the radiation-induced immunogenic cell death (ICD)^9^. DAMP molecules play a key role in the immunogenic potential to attract and activate dendritic cells (DCs) to phagocytose dying tumor cells, to process and present released tumor antigens to T cells^9,10^.

At present, there are no effective therapeutic strategies for the elimination of GSCs. Due to an enhanced repair capacity, GSCs recover rapidly from conventional therapeutic stress, which leads to resistance and eventual disease relapse in glioma patients. Augment of RT-induced endoplasmic reticulum (ER) stress might block self-recovery of GSCs and make cells to die. As a broad specificity molecular chaperone within ER, binding immunoglobulin protein (Bip), also known as 78-kDa glucose regulated protein (GRP78), correctly folds nascent polypeptides and regulates the unfolded protein response (UPR) ensuring protection of the cell from denatured protein and reinforcing its anti-apoptotic role, when the cell is under stress^11^. In addition, Bip is responsible for maintaining “stemness” in cancer cells^12,13^. To demonstrate the mechanism of GSCs resistance to IR-induced ICD, the role of Bip was evaluated in ER stress-activated ICD. In this study, we found high-dose ionizing radiation (IR) triggered fewer DAMPs molecules exposure and release comparing to non-GSCs, which made the immune response elicited by RT insufficient to eliminate GSCs. Bip inhibition efficiently enhanced ER stress and promoted IR-mediated DAMP molecules exposure and release in GSCs. These data suggested that promoting GSCs ICD should be a promising strategy to prevent or delay post-radiotherapy recurrence of GBM.

## Results

### IR induces less DAMP molecules exposure and release in GSCs comparing to non-GSCs

**Fig. 1:**
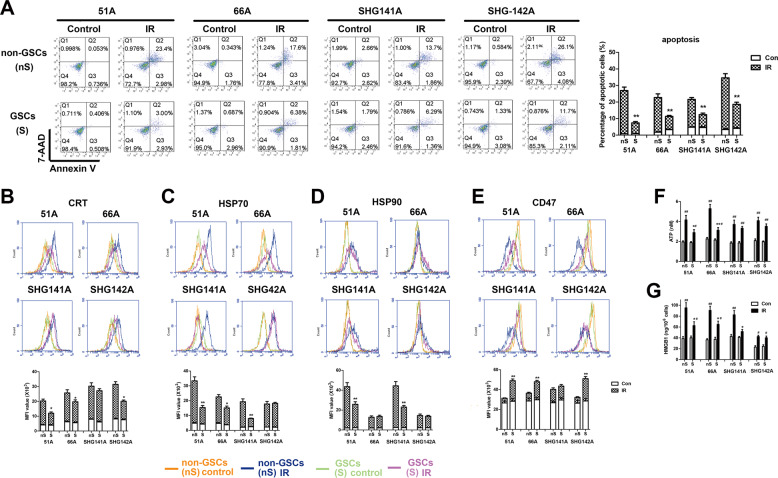
IR induces less cell apoptosis, immunogenic molecule exposure and release in GSCs compared with non-GSCs. GSCs and non-GSCs were incubated for 24 h after 10 Gy IR for apoptosis assay and detection of CRT, HSP90, HSP70, CD47, ATP, and HMGB1. **a** Cell apoptosis was determined by Annexin V and 7-AAD staining. The expressions of CRT (**b**), HSP70 (**c**), HSP90 (**d**), and CD47 (**e**) on cell surface were measured by flow cytometry. The level of extracellular ATP (**f**) using a chemiluminescence assay and HMGB1 release (**g**) in supernatant using an elisa assay were detected. *P < 0.05, **P < 0.01 vs matched non-GSCs, ^#^*P* < 0.05, ^##^*P* < 0.01 vs control.

The results of Annexin V and 7-AAD stain showed that less cell apoptosis was induced in GSCs comparing to non-GSCs after 10 Gy IR (Fig. 1a). It has been shown that IR triggers ICD in cancer cells^14–16^. Emission of ICD hallmark molecules from non-GSCs was significantly increased following 10 Gy IR (Supplementary Fig. 1). Next, we analyzed whether ICD can be induced by the same dosage of IR in cancer stem cells. The data showed less cell surface exposure of CRT, heat shock protein (HSP) 70 and HSP90 in all detected GSCs compared with non-GSCs except for CRT in SHG141A, HSP70 in SHG142A and HSP90 in 66A and SHG142A (Fig. 1b–d). CD47 expression on cell surface was significantly decreased after IR in both GSCs and non-GSCs, and less decrease on CD47 expression in GSCs comparing to matched non-GSCs were observed except for SHG141A (Fig. 1e).

We further detected the release of ATP by chemoluminescent assay and HMGB1 using Elisa assay. We observed the release of ATP (Fig. 1f) and HMGB1 (Fig. 1g) produced by IR from all detected GSCs and non-GSCs. IR-induced less ATP release in supernatant of 51A and 66A GSCs and less HMGB1 release in 51A, 66A and SHG141A GSCs compared with matched non-GSCs.

### Bip inhibition increased IR-induced ER stress and ICD in GSCs

Emerging evidences demonstrate the exposure and release of ICD hallmark molecules through ER stress^9^, which causes an overload and misfold of proteins in the ER^11^. To demonstrate whether stemness maintenance resulting from Bip upregulation gave rise to less DAMPs exposure and release in GSCs, specific ICD molecules 24 h following 10 Gy IR were detected when GSCs were pretreated with Bip inhibitor or siRNA-Bip transfection.

**Fig. 2:**
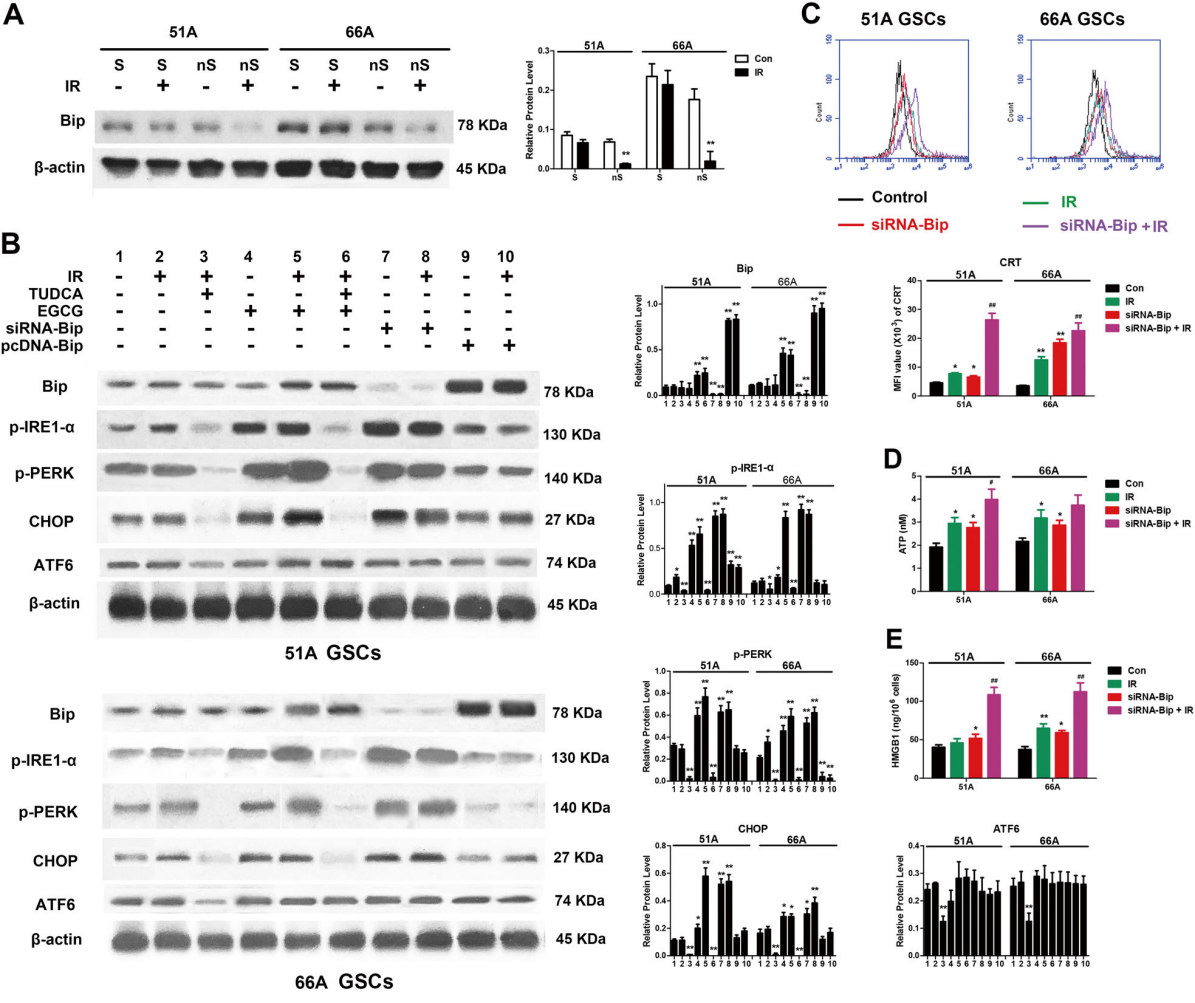
Bip inhibition promoted IR-mediated ER stress and ICD generation. **a** Bip expression was detected using western blot in GSCs and non-GSCs after cells were irradiated with 10 Gy. **b** EGCG or siRNA-Bip enhanced the increase of IR-induced ER stress associated protein in GSCs. GSCs were transfected with siRNA-Bip or pcDNA-Bip. After pretreatment with or without 2.5 mM TUDCA for 6 h, cells were irradiated with 10 Gy, then indicated protein expressions were detected by western blot. **c** The level of CRT exposure on cell surface was measured using flow cytometry, and MFI was analyzed. Cells were transfected or untransfected with siRNA-Bip following 10 Gy radiation. **d** The level of extracellular ATP was measured using a chemiluminescence assay. **e** HMGB1 in supernatant was detected using an elisa assay. *P < 0.05, **P < 0.01 vs control, ^#^*P* < 0.05, ^##^*P* < 0.01 vs IR.

We first found significant upregulation of Bip expression in all detected GSCs comparing to non-GSCs (Supplementary Fig. 2), and downregulation of Bip expression was showed postirradiation in non-GSCs but not GSCs (Fig. 2a). As shown in Fig. 2b, we next found the expression levels of ER stress associated protein were upregulated in GSCs 51A and 66A post-IR. Pretreatment with tauroursodeoxycholic acid (TUDCA), a chemical chaperone for blocking ER stress, of 2.5 mM for 6 h effectively inhibited expression of ER stress associated protein. To measure whether Bip downregulation can promote ER stress in GSCs after IR, a Bip inhibitor, epigallocatechin gallate (EGCG, Sigma Aldrich)^17^, was used to inhibit protein synthesis. The results showed that expressions of ER stress associated protein were significantly upregulated by 50 μM EGCG pretreatment for 24 h and more obvious following IR (Fig. 2b and Supplementary Fig. 3). EGCG showed similar function on increasing expressions of ER stress associated protein as Bip knockdown (siRNA-Bip transfection in GSCs) following IR. The results suggested Bip inhibition or knockdown efficiently triggered IR-mediated ER stress in GSCs.

Transfection with siRNA-Bip increased the exposure of CRT on cell surface (Fig. 2c), increased extracellular ATP level (Fig. 2d) and HMGB1 release (Fig. 2e) induced by IR in 51A and 66A GSCs, suggesting the increase of IR-induced ICD hallmark molecules by Bip inhibition in GSCs. Meanwhile, Bip overexpression decreased the change of IR-induced ICD hallmark molecules in non-GSCs (Supplementary Fig. 4).

### Bip inhibition enhances IR-mediated PERK and IRE1-α activation

The mechanism of ICD induction is associated with ER stress, a conserved cellular program that tries to cope with UPR resulting from dysfunctions^18^. In normal cells, the UPR is regulated by three major pathways involving activating protein kinase RNA-like ER kinase (PERK), inositol-requiring protein 1-α (IRE1-α) and activating transcription factor 6 (ATF6)^18^.

**Fig. 3:**
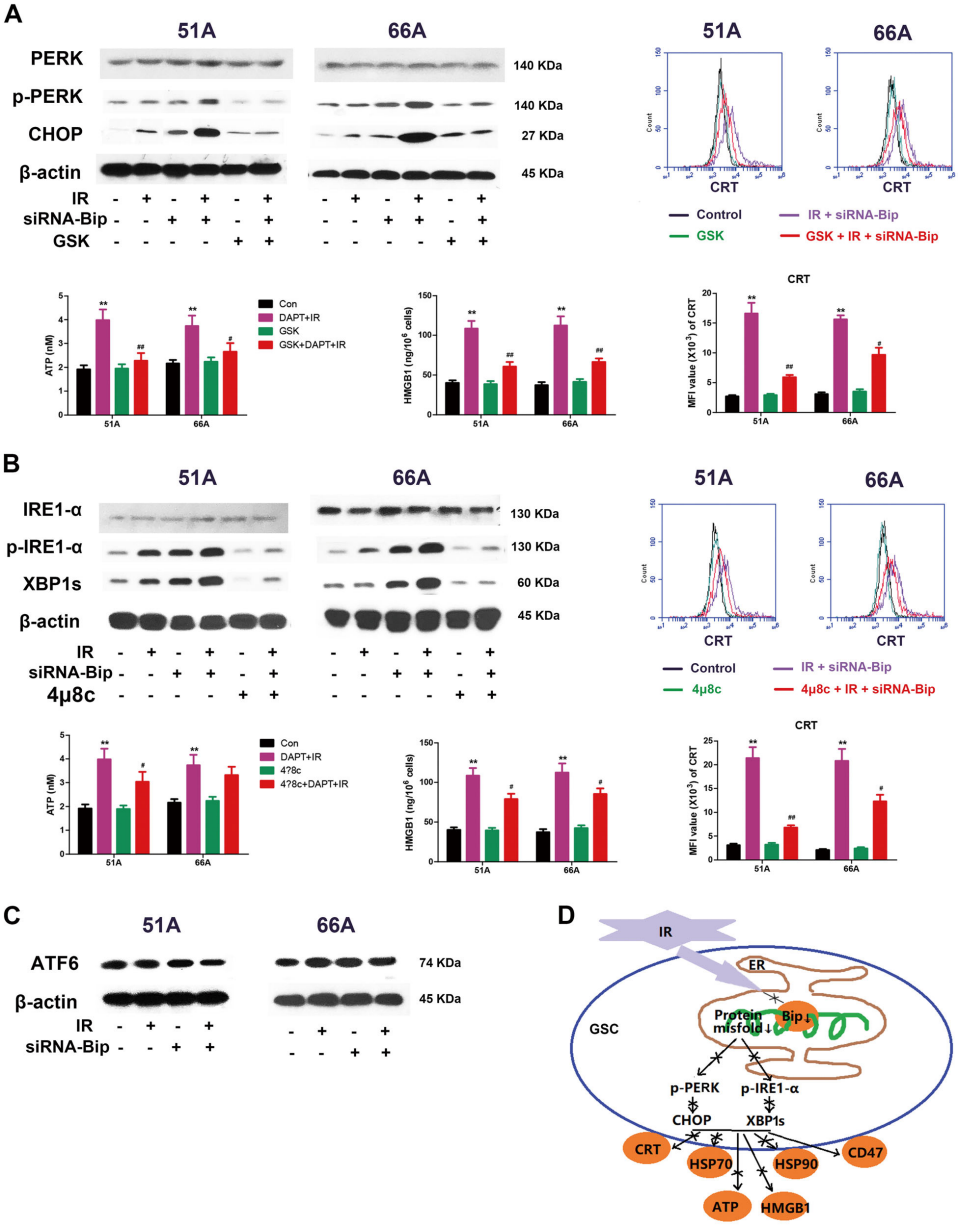
Bip knockdown enhances the activation of IR-induced specific UPR branch pathways. GSCs transfected or untransfected with siRNA-Bip were treated with IR 10 Gy in the presence or absence of UPR pathway inhibitors, then UPR proteins and DAMPs were detected at 24 h after irradiation. **a** The changes of PERK pathway proteins and DAMPs were analyzed with or without pretreatment with 2 μM GSK2606414, a specific PERK pathway inhibitor, for 1 h before irradiation. **b** The expressions of IRE1-α pathway proteins and DAMPs were measured in presence or absence of 10 μM 4u8c, a specific IRE1-α pathway inhibitor, for 1 h before irradiation. **c** Protein level of ATF6 was determined by immunoblot. **d** The mechanism of IR-mediated decreased ICD in GSCs. *P < 0.05, **P < 0.01 vs control, ^#^*P* < 0.05, ^##^*P* < 0.01 vs IR.

For PERK pathway analysis, we observed no increased phosphorylated PERK expression following irradiation in 51A and 66A GSCs. Transfection with siRNA-Bip before IR significantly upregulated the expression of phosphorylated PERK and CHOP, relative to IR treatment alone, using western blot assay (Fig. 3a). To examine the role of the PERK pathway in Bip inhibition-enhanced UPR in GSCs, GSK2606414, a specific pharmacological inhibitor of PERK phosphorylation, was used. When GSCs were treated with GSK2606414+IR+siRNA-Bip, reduction in the levels of phosphorylated PERK was accompanied by a significant decrease in CRT exposure on cell surface, extracellular ATP level and HMGB1 release, indicating the involvement of PERK signaling in Bip-inhibited immunogenic death (Fig. 3a).

For IRE1-α pathway analysis, the expression of phosphorylated IRE1-α in 51A and 66A GSCs and splicing XBP1 (XBP1s) in 51A GSCs was upregulated following irradiation. SiRNA-Bip transfection further enhanced the expression of phosphorylated IRE1-α and downstream XBP1s following irradiation (Fig. 3b). To investigate the role of IRE1-α pathway in GSCs with Bip knockdown and IR treatment, we used 4μ8c, a specific inhibitor of IRE1-α. Pretreatment with 4μ8c showed a significant decrease in phosphorylated IRE1-α and XBP1s protein coupled with reduction in CRT exposure, extracellular ATP level and HMGB1 release in IR and siRNA-Bip treating cells. These finding suggested that IRE1-α pathway was also involved in ICD induced by IR and Bip inhibition (Fig. 3b).

For ATF6 pathway analysis, we found no difference in cleavage of ATF6 in GSCs with or without Bip knockdown after irradiation. We could exclude the participation of ATF6 pathway activation in anti-ICD efficacy of Bip upregulated GSCs. (Fig. 3c).

Together, these findings advocate that IR can promote partial UPR protein expression in GSCs, and the Bip inhibition enhances radiation-induced ICD, which is mediated by ER stress and is specifically dependent on PERK and IRE1-α pathways (Fig. 3d).

### Bip inhibition elevates phagocytosis of DCs on IR-treated GSCs

**Fig. 4:**
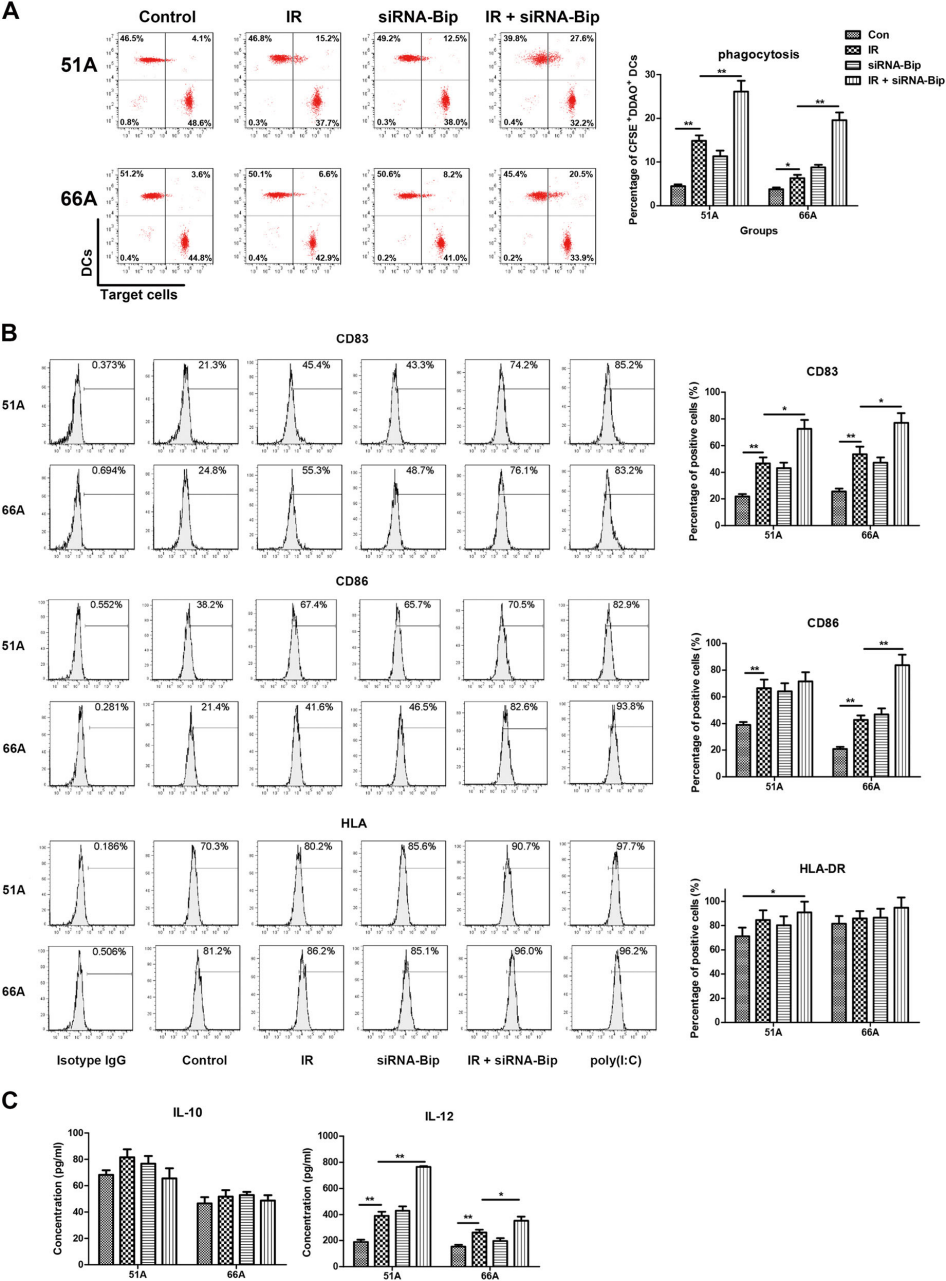
ICD induced phagocytosis of IR-treated GSCs, DCs phenotypic maturation, cytokine production. DCs were generated from PBMCs of three healthy doors after culture of adherent monocytes with rhGM-CSF and rhIL-4 for 7 days. GSCs transfected with or without siRNA-Bip were irradiated with 10 Gy and incubated for 24 h, then were added to human DCs at ratio 1:1 and incubated for additional 24 h, then the characteristics of DCs were determined. **a** The percentage of DCs positive for CFSE dye after co-culture with GSCs for far red dye was assessed by flow cytometry, and the percentage of double positive cells (DCs/GSCs) was calculated. **b** Specific markers expression of DCs maturation was evaluated by flow cytometry using monoclonal antibodies. **c** The levels of IL-10 and IL-12 was examined from cell culture supernatants by elisa. ^#^*P* < 0.05, ^##^*P* < 0.01.

Dying tumor cells with CRT translocation on cell surface were engulfed by dendritic cells (DCs)^19^. We analyzed whether Bip knockdown and IR-induced ICD on GSCs would impact on phagocytosis of DCs. As shown in Fig. 4a, the percentage of DCs phagocytosis is (26.1 ± 2.5)% in 51A and (19.6 ± 1.8)% in 66A GSCs in combination of siRNA-Bip transfection and IR treatment.

The capability of IR and/or siRNA-Bip transfection treated cells to induce the maturation of monocyte-derived DCs was examined by flow cytometry. Phagocytosis of IR-treated cells yielded a mature-DC phenotype of CD83 and CD86 as compared to control, and combination of IR and siRNA-Bip transfection increased DCs maturation, which was evidenced by increased expression of specific markers including CD83, CD86 and HLA, except for CD86 expression in 51A GSCs and HLA expression (Fig. 4b). Isotype IgG or poly(I:C) stimulation was used as negative or positive control, respectively. Transfection with siRNA-Bip increased the production of an important cytokines related to DCs function, IL-12, from DCs after IR in detected cells, however, the level of IL-10 showed no obvious change (Fig. 4c). These data indicate that ICD-induced DCs phagocytosis, specific phenotypic maturation and cytokine secretion of IR-treated GSCs are increased by Bip knockdown.

### Bip downregulation of GSCs is critical for activation of effector T lymphocyte

**Fig. 5:**
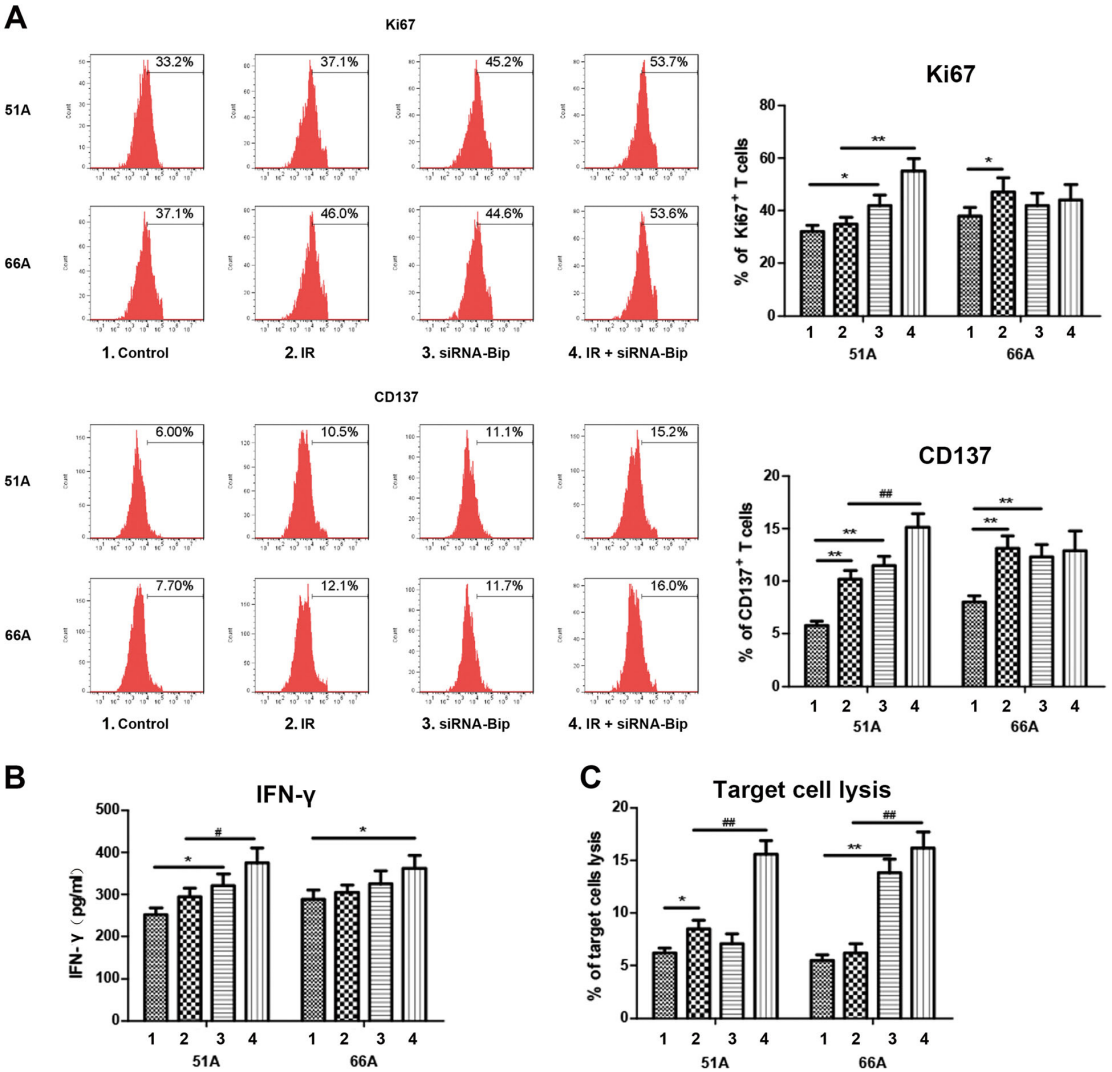
Effector T lymphocyte activation were notably occurred in GSCs of combinative treatment with IR and Bip knockdown. DCs were co-cultured with autologous isolated T lymphocytes after pulsed with siRNA-Bip- and/or IR-treated GSCs. Activated T lymphocytes as effector cells were re-stimulated with target GSCs, then Ki67^+^ and CD137^+^ T lymphocytes using flow cytometry and IFN-γ secretion in supernatant using elisa were detected. Target cells were labeled with CFSE, and co-cultured with unlabeled T lymphocytes pulsed by DCs. Lysis of target cells was measured using 7-AAD stain. The percentage of Ki67^+^ and CD137^+^T lymphocyte (**a**), IFN-γ secretion in supernatant (**b**) and percentage of target cell lysis (**c**) were analyzed after target cells were added to DCs-activated T lymphocyte.

T lymphocyte stimulatory capacity of DCs pulsed with siRNA-Bip and/or IR-treated cells was evaluated after T cells were co-incubated with the target GSCs. The results were presented in Fig. 5. Combination of siRNA-Bip transfection and IR treatment resulted in more Ki67^+^ and CD137^+^ T cells in 51A GSCs as target cells (Fig. 5a), a significantly higher production of IFN-γ in 51A GSCs (Fig. 5b), and an elevated cell lysis in 51A and 66A GSCs (Fig. 5c) compared with IR alone. These data again indicating that Bip inhibition triggered a more potent anti-glioma immune response compared with IR alone.

### GSCs transfected with Bip siRNA following IR as a vaccine prevent efficiently from tumor generation

**Fig. 6:**
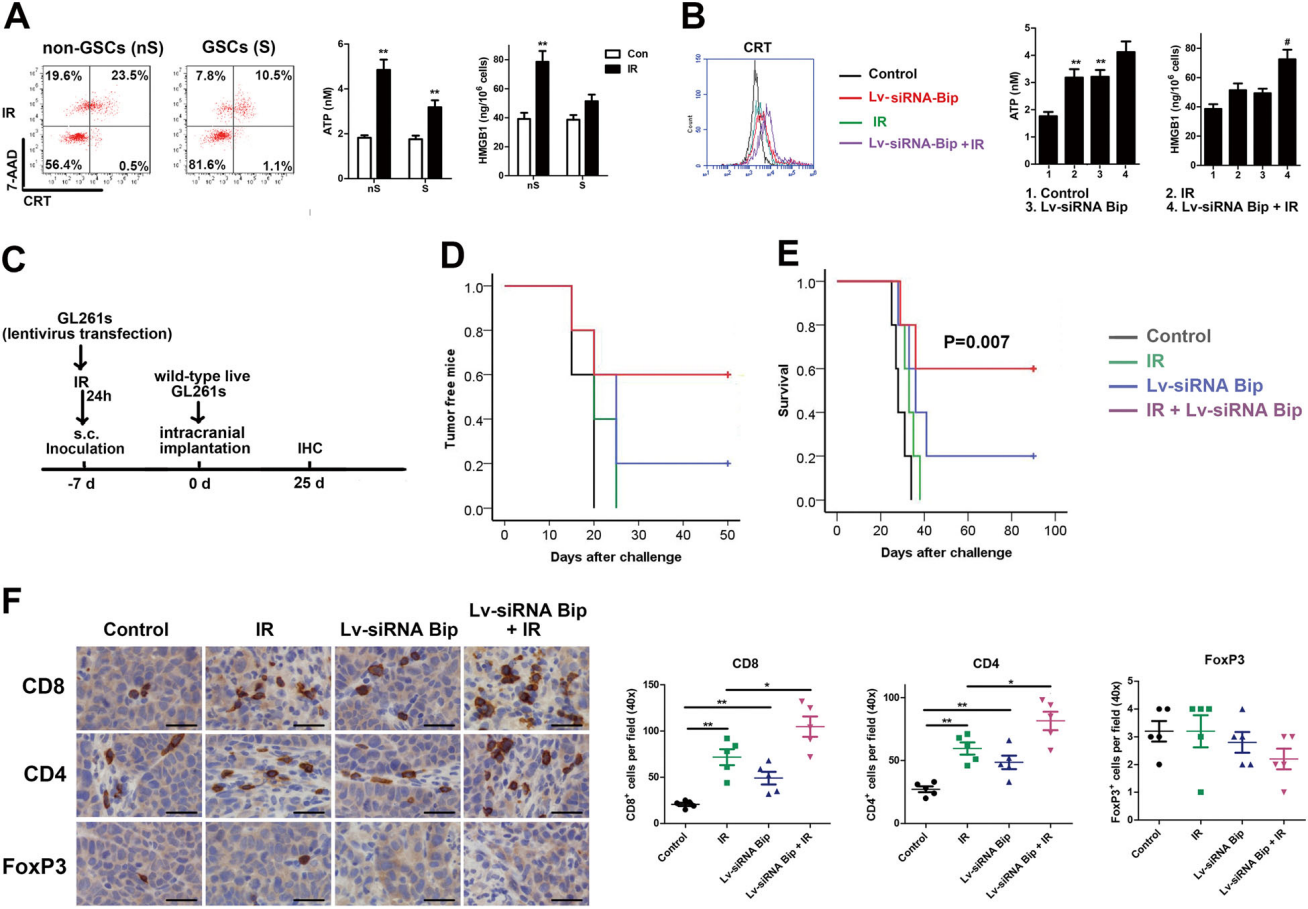
GSCs treated with Bip inhibition following high-dose IR as a vaccine prevent efficiently tumor generation. GL261s GSCs with lentivirus transfection following 10 Gy IR were inoculated subcutaneously into the left flank of C57BL/6J mice, then untreated live cells were intracranially implanted into the mouse cerebrum 7 days later. **a** IR induces ICD hallmark molecules exposure in mouse GSCs and non-GSCs in vitro. Mouse glioma cell line GL261 and GSCs GL261s were irradiated with 10 Gy, CRT on cell surface, ATP and HMGB1 release in supernatant were measured 24 h after IR treatment. **b** Bip knockdown promoted IR-mediated ICD in mouse GSCs GL261s. GL261s transfected or untransfected with siRNA-Bip lentivirus were irradiated with 0 or 10 Gy, ICD hallmark molecules were detected 24 h later. *P < 0.05, **P < 0.01 vs control, ^#^*P* < 0.05, ^##^*P* < 0.01 vs IR. **c** The schedules of preventing treatment were indicated. **d** Tumor-free mice were calculated after live GL261s implantation. **e** Survival curves of C57BL/6 mice was analyzed after live GL261simplantation. **f** Immune subsets were showed using anti-CD4, CD8 and FoxP3 antibodies in intracranial allografts 25 days after cell implantation. Average number of stained cells were showed. Scale bar = 50 μm, *P < 0.05, **P < 0.01.

Less ICD hall markers were showed after mouse GSCs GL261s comparing to matched non-GSCs GL261 were irradiated at a dose of 10 Gy (Fig. 6a). Stable transfection with siRNA-Bip lentivirus significantly enhanced the production of DAMPs molecules in GL261s (Fig. 6b). To estimate whether high-dose-IR and Bip inhibition-treated cells have an effect of preventing the generation of glioma from GSCs as a vaccine in vivo, the syngenic C57BL/6J mice were administrated with lentivirus transfection- and/or IR-treated GL261s GSCs subcutaneously before untreated GL261s GSCs were implanted intracranially for tumor model establishment. As shown in Fig. 6d, e, pretreatment with siRNA-Bip- and IR-treated GL261s GSCs was highly efficient in preventing the growth of live GL261s injected 7 days later. The efficacy of vaccination from IR-treated GL261s was attenuated without Bip-siRNA transfection. These results confirmed that GSCs with Bip inhibition following IR as a vaccine could prevent tumor generation efficiently.

Next, immune cell subset were evaluated by IHC stain on formalin fixed paraffin embedded (FFPE) tissues (Fig. 6f). Increase of CD4^+^ and CD8^+^ T cells were observed in the group of Lv-siRNA-Bip transfection and IR combination comparing to other treatment groups, indicating the cardinal role of Bip in facilitating high-dose irradiation induced immunogenicity.

### Bip inhibition- and IR-induced immunity has a significantly adjuvant antitumor efficacy to radiochemotherapy in vivo

**Fig. 7:**
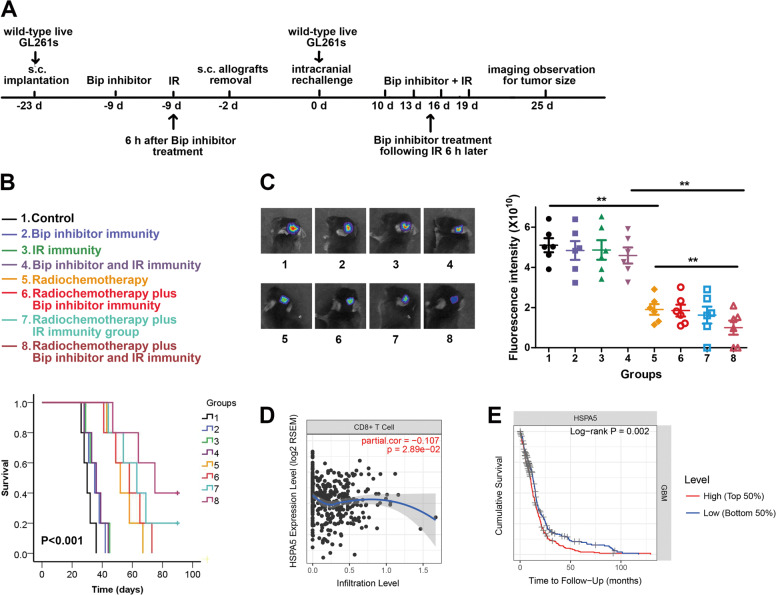
Bip inhibition and IR immunity following radiochemotherapy have a significant antitumor efficacy, and Bip expression is relative to immune infiltration. C57BL/6J mice were subcutaneously implanted with untreated GL261s, then the allografts were irradiated and treated with Bip inhibitor. After removal of primary tumors, the mice were intracranially implanted with untreated live GL261s cells. **a** The schedules of therapeutic regimen were indicated. **b** Survival curves of mice was analyzed under indicated therapeutic regimen. **c** Tumor size were measured after live GL261s rechallenge. **d** Bip gene expression has a negative correlation with CD8^+^ T cell infiltration. **e** Bip gene expression and GBM patients’ survival showed a negative correlation. *P < 0.05, **P < 0.01.

To determine whether the immunity induced by high-dose-IR and Bip inhibition refuses tumor rechallenge and promotes antitumor efficacy of radiochemotherapy, the s.c. allografts of C57BL/6J mice were treated with Bip inhibitor and 10 Gy irradiation, then primary tumors were resected, and mice were rechallenged intracranially with live GL261s (Fig. 7a). Radiochemotherapy plus Bip inhibitor and IR-induced immunity efficiently extended survival of glioma bearing mice (Fig. 7b), and reduced tumor sizes after GL261s rechallenge (Fig. 7c). These results suggested the immunity induced by high-dose irradiation and Bip inhibition promoted antitumor efficacy of radiochemotherapy.

### The relative analysis of Bip expression and immune cells infiltration in GBM patients

We analyzed the relationship of Bip gene (HSPA5) and immune cells infiltration in glioma patients using the website of Tumor Immune Estimation Resource (https://cistrome.shinyapps.io/timer/). The analyzed result showed that Bip expression was negative correlation with CD8^+^ T cell infiltration (Fig. 7d) and GBM patients’ survival (Fig. 7e), consistent with our results in vitro and animal models.

## Discussion

GBM is well-known to contain few immune cells in its microenvironment^19^. Immunotherapy for glioma malignancies has met significant challenges. Research results are inconsistent about whether neoadjuvant PD-1 regimen significantly improved both overall and progression-free survival of GBM patients in clinical trials^20,21^. However, more immune cell infiltration was observed in long-term responders, supporting a local immuno-modulatory effect of treatment^21^.

The biologic features of stem cells are relevant to immune therapies, since stem cells are essential for the repair of tissue damage^22^. In this study, we analyzed the mechanism of GSCs resistance to RT-induced immune response to protect themselves from immune damage, and found the notable correlation of GSCs and IR-induced ICD. To promote DAMP molecules exposure and release following IR in GSCs, the knockdown of Bip, an ER stress sensor, was designed to enhance IR-induced ICD. Our results demonstrated that Bip downregulation elicited apoptosis, ER stress and DAMPs molecules exposure on cell surface and extracellular release. The phagocytosis and maturation of DCs and the proliferation of T lymphocytes following IR were augmented when GSCs were transfected with siRNA-Bip. Combination of Bip inhibition and IR increased CD4^+^ and CD8^+^ immune cells and decreased Treg cells infiltration in vivo. Notable prevention function was showed using Bip knockdown- and IR-treated GSCs as a vaccine, and tumor delayed growth and longer survival were showed following Bip inhibition treatment and local IR far from tumor implantation region.

A new strategy suggests a combination of immune intervention and local irradiation for solid tumor, due to irradiated tumors triggering a better antitumor immune response (6–8). Previous studies showed that cancer cells exposed to a high dose of RT^14^ or chemotherapy reagent in vitro, such as mitoxantrone^23^, oxaliplatin^24^, and doxorubicin^25^, were capable of vaccinating syngeneic mice against a subsequent challenge with living cells of the same type. In this study, we demonstrated fewer DAMPs were exposed and released in IR-treated GSCs, resistant to RT and chemotherapy, compared with non-GSCs. Less ICD generation becomes one mechanism of GBM recurrence. New regimens for promoting GSCs ICD may be efficient to inhibiting GBM recurrence.

ER stress initially serves as an adaptive measure to protect the cell from irreversible damage in cancer^11^. In theory, increased Bip reduces protein misfold in GSCs to maintain GSCs growth and stemness characteristics. Our results in western blot showed higher Bip expression levels in GSCs comparing to non-GSCs, which was consistent with previous studies^12,13^. We hypothesized that knockdown of Bip augmented misfold of nascent polypeptides, which would induce ER stress and UPR, and trigger GSCs ICD. In order to verify whether fewer DAMPs release was associated with high Bip expression, siRNA-Bip was used to investigate the change of IR-induced GSCs ICD. Our results showed Bip knockdown increased IR-induced apoptosis, the translocation of CRT, HSP70 and HSP90, the release of ATP and HMGB1, and decreased CD47 expression on cell surface, suggesting IR-induced ICD enhancement by Bip inhibition.

The accumulation of misfolded polypeptides in the ER lumen results in UPR, which is involved in the molecular mechanisms underlying ICD hallmark^18^. Our results showed Bip inhibition activated two of three UPR signal pathways induced by IR, including PERK and IRE1-α pathways, in radioresistant GSCs. PERK activated by combined treatment of Bip inhibition and IR was phosphorylated and selectively enhanced CHOP expression. Similarly, combination of Bip inhibition and IR treatment dissociated IRE1-α from BiP, and IRE1-α underwent dimerization and autophosphorylation, then activated and spliced the target, XBP1. The results were consistent with the mechanism of BiP binding to the ER luminal domains, preventing homodimerizatio^26^. In the presence of cellular stress, accumulation of misfolded proteins within the ER bound BiP competitively, causing dissociation of BiP from PERK and IRE1-α, thus removing its inhibitory effects^26^.

## Materials and methods

### Primary cell culture of human GSCs and cell lines

GSCs 51A and 66A are from GBM patients, as gifts from Professor Yihong Zhou at the UC Irvine Brain Tumor Research Laboratory. GSCs SHG141A and SHG142A were isolated from GBM surgical specimen in the First Affiliated Hospital of Soochow University. GSCs were cultured in DMEM/F12 medium that was supplemented with 10% fetal bovine serum (FBS) to induce differentiation for one month, considering as non-GSCs. Mouse glioma cell line GL261 were cultured in high glucose DMEM with 10% FBS. Then these cells were cultured in stem cell medium for 12 passages to produce GSCs GL261s, and cells were used as GSCs after identification. All cells are isolated, cultured and identified as previously described^27^.

### Ionizing radiation

The cells and s.c. allografts of mice were exposed to 10 Gy X-ray (160 kV) at room temperature using a linear accelerator (RadSource, Suwanee, GA, USA) at a dose rate of 0.50 Gy/min. Radiotherapy on intracranial allografts of mice was subjected to 8 Gy (2 Gy/d once every 3 days for 4 times) X-ray irradiation (6 MV, the dose rate was 100 cGy/min) by a PRIMUS accelerator (SIEMENS Medical Solutions, Erlangen, Germany) at room temperature. Irradiation was locally confined to the tumors by shielding the rest of the body with lead.

### Flow cytometry

Neurospheres were dissociated into single cells, and adherent cells were trypsinized and collected. Cells were stained using FITC-Annexin V/7-AAD apoptotic Kit (Biolegend) according to the manufacturer’s instructions. Cells were incubated with antibodies for 30 min at room temperature. The primary antibodies include APC-conjugated nestin mouse antibody (1:10; Invitrogen), PE-conjugated CD133 antibody (1:11; Miltenyi Biotech GmbH), PE-conjugated anti-CRT rabbit antibody (1:50, Cell Signaling), PE-conjugated anti-HSP90 rabbit mAb (1:50, Cell Signaling), Alexa Fluor® 488-conjugated anti-HSP70 rabbit antibody (1:500, Abcam), FITC-conjugated anti-CD47 mouse antibody (1:20, eBioscience™), PE-conjugated anti-HLA-DR mouse antibody (1:20, Cell Signaling), PE-conjugated anti-CD83 mouse antibody (1:20, eBioscience™), PE-conjugated anti-CD86(B7-2) mouse antibody (1:20, eBioscience™), PE-conjugated anti-Ki67 rabbit antibody (1:50, Cell Signaling), PE-conjugated anti-CD137 rabbit antibody (1:50, Cell Signaling). Labeled cells were analyzed by a flow cytometic Beckton Dickinson FACScan (BD Biosciences). Data were analyzed using FlowJo Software version 7.0 and presented by mean fluorescence intensities (MFI) or positive cell number.

### Western blot analysis

Cell lysates were prepared in RIPA lysis buffer containing phenylmethylsulfonyl fluoride (PMSF). The protein samples were separated by 10% sodium dodecyl sulfate-polyacrylamide gel (SDS-PAGE), then were transferred onto the polyvinylidene fluoride (PVDF) membrane. The membrane was then blocked with 5% non-fat dry milk for 1 h. Primary rabbit antibodies include anti-Bip (1:1000, Cell Signaling), anti-CHOP (1:1000, Cell Signaling), anti-PERK (1:1000, Cell Signaling), phospho-PERK (1:1000, Invitrogen), IRE1-α (1:1000, Cell Signaling), p-IRE1-α (1:1000; Invitrogen), splicing XBP1 (1:1000, Cell Signaling), ATF6 (1:1000, Cell Signaling). HRP-conjugated anti-rabbit and anti-mouse secondary antibodies were used and the chemiluminescent signal was detected by using electrochemiluminescence (ECL) reagents (Invitrogen).

### ATP and HMGB1 assays

5 × 10^5^ cells following IR were incubated for 24 h, then extracellular ATP and HMGB1 release were measured. Extracellular ATP after IR was measured by an ATP Assay Kit (Promega, Madison, USA) based on luciferin–luciferase conversion following the manufacturer’s instructions. The chemoluminescent signal was read by a Synergy Neo2 Hybrid Multi-Mode Reader (BioTek, USA). HMGB1 in supernatant was determined by a HMGB1 ELISA kit (Biorbyt, UK) according to manufacturer’s instructions. The microplates were read using a multiskan spectrophotometer model 1510 (Thermo Fisher Scientific, Finland) for protein concentration assessment.

### SiRNA and plasmid transfection

For upregulation or downregulation of Bip, siRNA-Bip or pcDNA-Bip, purchased from Shanghai Genepharma Co., Ltd., was transfected into 51A and 66A GSCs using Lipofectamine 2000 (invitrogen) according to the manufacturer’s instructions. The cell were transfected with nontargeting control (NTC) siRNA as a control.

### DCs generation and maturation

Human peripheral blood mononuclear cells (PBMCs) from healthy donors were purified by Ficoll-Hypaque gradient centrifugation of heparinized blood. PBMCs were divided into two parts, one half was used for DCs culture, and the other half was frozen until they were used as effector cell production in later experiments. Monocyte-derived DCs were obtained from CD14^+^ peripheral blood monocytes isolated using magnetic beads (MiltenyiBiotec). Immature DCs (iDCs) were obtained by culturing for seven days in RPMI-1640 medium in presence of 10% FBS, 800 U/ml rhGM-CSF and 50 ng/ml rhIL-4. Immature DCs were stained with CellTrace^TM^ CFSE cell proliferation Kit (Invitrogen) for flow cytometry. GSCs were stained with CellTrace^TM^ Far Red cell proliferation Kit (Invitrogen). 1 × 10^6^ NTC, irradiated and/or siRNA-Bip transfected GSCs were added to iDCs in 1:1 ratio for 24 h culture for analysis of phagocytosis. DCs that phagocytosed GSCs were double positive cells of CFSE and far red using flow cytometry. Phenotypical assessment of DCs was determined by flow cytometry using fluorescein labeled monoclonal antibodies to HLA-DR, CD83 and CD86. iDCs were incubated with 25 μg/mL poly(I:C) (Polyinosinic–polycytidylic acid sodium salt, Sigma-aldrich) for 24 h as a positive control of DCs maturation. IL-10 (Human IL-10 Pre-Coated ELISA Kit, BioGems) and IL-12 (Human IL-12 (p70) Pre-Coated ELISA Kit, BioGems) concentrations in cell culture supernatants were determined according to the manufacturer’s instructions.

### T cell isolation, target cell proliferation, and lysis assays

T lymphocytes were purified by pan T cell isolation kit (MiltenyiBiotec GmbH) from PBMCs, and labeled by CFSE.DCs were pulsed with NTC, siRNA-Bip and/or IR-treated GSCs, then co-cultured with autologous isolated T lymphocytes at ratio 10:1 for 7 days. 50 U/mL IL-2 was added on days 3 and 5. T lymphocytes as effector cells for antitumor immune response study were re-stimulated with target GSCs (1 × 10^4^ cells in 100 μl) treated with original samples at an effector-to-target ratio (E:T) ratio of 10:1 for 12 h. Flow cytometry detected Ki67^+^ and CD137^+^ T lymphocytes, and Elisa assay was used for IFN-γ secretion in supernatant. Lysis of target cells was measured using flow cytometry. Target cells were labeled with far red, and co-cultured in a 96 U-bottom well plate with T lymphocytes pulsed by DCs. Dead cells were detected by flow cytometry using 7-AAD stain.

### Generation of stable cell lines

GL261s GSCs with stable integration of Bip cDNA and Bip-siRNA sequences were generated through lentiviral-mediated gene transfection (Shanghai GenePharma Co., Ltd., Shanghai, China) according to the manufacturer’s instructions. More than 90% of GSCs with the stably integrated gene were considered GSCs of Bip overexpression or Bip knockdown.

### Antitumor vaccination

The male syngenicC57BL/6J mice about 18–20 g were bred and housed in a specific pathogen free condition. All animal experimental protocols were approved by the Institutional Animal Care and Use Committee of Soochow University and complied with the code of ethics for animal experimentation.

Twenty mice were randomly divided into four groups including control (transfection with scramble lentivirus), IR (transfection with scramble lentivirus following IR), Lv-siRNA-Bip (transfection with siRNA-Bip lentivirus) and IR plus Lv-siRNA-Bip (transfection with siRNA-Bip lentivirus following IR). GL261s GSCs stably transfected with lentivirus were irradiated with 10 Gy, then cultured for 24 h. 1 × 10^7^ treated GL261s cells were inoculated subcutaneously into the left flank of male syngenic C57BL/6J mice as a vaccine. Whereas 1 × 10^5^ untreated live cells were intracranially implanted into the frontal lobe of the mouse cerebrum by stereotactic implantation to establish intracranial transplantation 7 days later. In vivo imaging system (IVIS) 50 system was used to monitor the allografts at day 15 and day 20 after intracranial implantation with live GL261s GSCs. Mice were monitored daily until severe neurological deficits appeared. No neurological deficit syndrome appearance was considered tumor-free mice at 50 day. The indicated treatment schedules were given in Fig. 6c. Survival analysis was used to compare the differences of each group according to survival time.

### Antitumor therapy

Flank tumors were established by subcutaneous injection of 5 × 10^6^ live GL261s GSCs suspension in a volume of 100 μL into the left flank. C57BL/6J mice with subcutaneous tumor were randomly divided into 8 groups: (1) Control group, no treatment; (2) Bip inhibitor immunity group, Bip inhibitor was administrated when subcutaneous tumor formation; (3) IR immunity group, subcutaneous tumor was irradiated; (4) Bip inhibitor and IR immunity group, the mice was administrated with combination of Bip inhibitor and irradiation treatment when subcutaneous tumor formation; (5) radiochemotherapy group, the mice was treated with fractionated radiation and Bip inhibitor after rechallenge with GL261s GSCs; (6) radiochemotherapy plus Bip inhibitor immunity group, the mice was treated with Bip inhibitor after subcutaneous tumor formation, then fractionated radiation and Bip inhibitor after intracranial GSCs rechallenge; (7) radiochemotherapy plus IR immunity group, allograft was irradiated after subcutaneous tumor formation, then the mice was treated with fractionated radiation and Bip inhibitor after rechallenge; 8. Radiochemotherapy plus Bip inhibitor and IR immunity group, the mice was administrated with combination of Bip inhibitor and irradiation treatment when subcutaneous tumor formation, then fractionated radiation and Bip inhibitor after rechallenge.

Bip inhibitor EGCG was dosed at 25 mg/kg in a volume of 200 μL by intraperitoneal (i.p.) injection 15 days after GL261 GSCs inoculation. Focal radiation was delivered in one fraction of total 10 Gy at 6 h after Bip inhibitor treatment. Seven days postirradiation, subcutaneous tumors were removed, and two days later mice were rechallenged intracranially with 1 × 10^5^ untreated live GL261s GSCs. The mice were administrated i.p. injection of Bip inhibitor at 25 mg/kg on day 10, 13, 16 and 19 and local IR 6 h later with the dose of 2 Gy after intracranial rechallenge. Tumor size was visualized by IVIS image on intracranial postimplantation day 25, and survival time was recorded for survival analysis. The indicated treatment schedules were given in Fig. 7a.

### Immunohistochemical (IHC) staining

Tumor tissues were fixed in paraffin, imbedded and cut for 4 mm sections. Tumor sections were incubated with primary antibodies, including anti-mouse CD4 (1:100, Cell Signaling), CD8 (1:400, Cell Signaling) and FoxP3 (1:100, Cell Signaling) antibodies, at 4 °C overnight, and biotin-labeled secondary antibody for 30 min at 37 °C. The final signal was developed using the 3,3′-diaminobenzidine (DAB) substrate and the sections were observed under optical microscope.

### Statistical analysis

All samples in vitro were carried out in triplicate independent experiments and represented as mean ± SD using GraphPad Prism 5 software. Data were statistically determined by one-way ANOVA, and the significance level was considered at a value of *P* < 0.05. Overall mouse survivals were estimated via Kaplan–Meier survival curves using SPSS software version 19.0.

## Conclusions

Together, these findings supported the notion that inhibition of Bip fostered the switch from IR-induced ICD-resistant GSCs to ICD-sensitive GSCs by activating UPR pathway. Improved immunogenicity of GSCs could facilitate T lymphocytes recognition and overcome therapy resistance. Enhancing immune response in GBM microenvironment by facilitating DAMPs exposure and release in adjuvant therapy makes it a promising strategy to prevent tumor generation and inhibit tumor recurrence.

## Supplementary information

Supplementary Figure legend

Supplementary Figure 1

Supplementary Figure 2

Supplementary Figure 3

Supplementary Figure 4
