# Supplementary material for: Bip inhibition in glioma stem cells promotes radiation-induced immunogenic cell death
Source: Cell Death Dis. 2020 Sep 22;11(9):786. doi: 10.1038/s41419-020-03000-z (PMC7508950; doi:10.1038/s41419-020-03000-z)
Supplement: Supplementary file 1 — Supplementary Figure legend [file 41419_2020_3000_MOESM1_ESM.docx]

**Supplementary Figure Legend**

**Supplementary Figure. 1 Different dosage IR induced emission of ICD hallmark molecules in non-GSCs.** The expression of CRT (A), extracellular ATP (B) and HMGB1 release (C) were detected in 51A non-GSCs (51A nS) and 66A non-GSCs (66A nS) following 0, 4, 6, 8, 10 and 12 Gy IR. **P* < 0.05; ***P* < 0.01 vs 0 Gy.

**Supplementary Figure. 2 Bip expression was detected using western blot in GSCs and non-GSCs.** **P* < 0.05; ***P* < 0.01 vs S.

**Supplementary Figure. 3 EGCG, a Bip inhibitor, has cytotoxic effects on GSCs.** GSCs were seeded in 96-well plates, and treated at the indicated concentrations of EGCG. Cell proliferation was assessed using a 3-(4,5-dimethylthiazol-2-yl)-2,5-diphenyl tetrazolium bromide (MTT) assay at 48 h. Optical density was recorded using a microplate reader at 570 nm. Proportions of proliferating normalised to controls are shown. **P* < 0.05; ***P* < 0.01 vs concentration of 0 μM.

**Supplementary Figure. 4 Bip overexpression decreased the change of IR-induced ICD in non-GSCs.** Non-GSCs were irradiated with 10 Gy, then ICD hallmark molecules were detected including CRT exposure on cell surface (A), extracellular ATP (B) and HMGB1 in supernatant (C). **P*<0.05, ***P*<0.01 vs control, ^#^*P*<0.05, ^##^*P*<0.01 vs IR.
