# Supplementary figures and images for: Bip inhibition in glioma stem cells promotes radiation-induced immunogenic cell death
Source: Cell Death Dis. 2020 Sep 22;11(9):786. doi: 10.1038/s41419-020-03000-z (PMC7508950; doi:10.1038/s41419-020-03000-z)

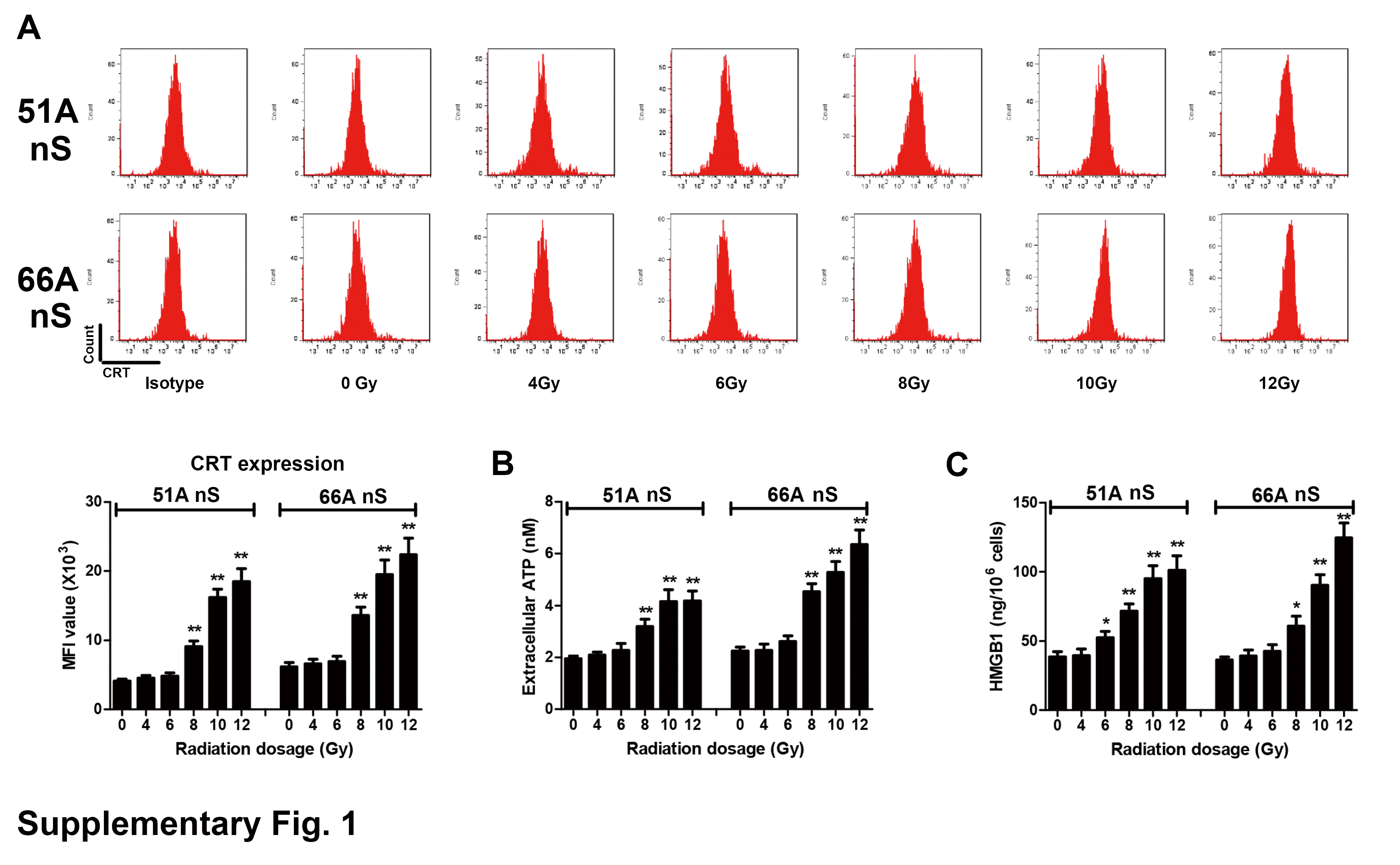

Supplement: Supplementary file 2 — Supplementary Figure 1 [file 41419_2020_3000_MOESM2_ESM.jpg]

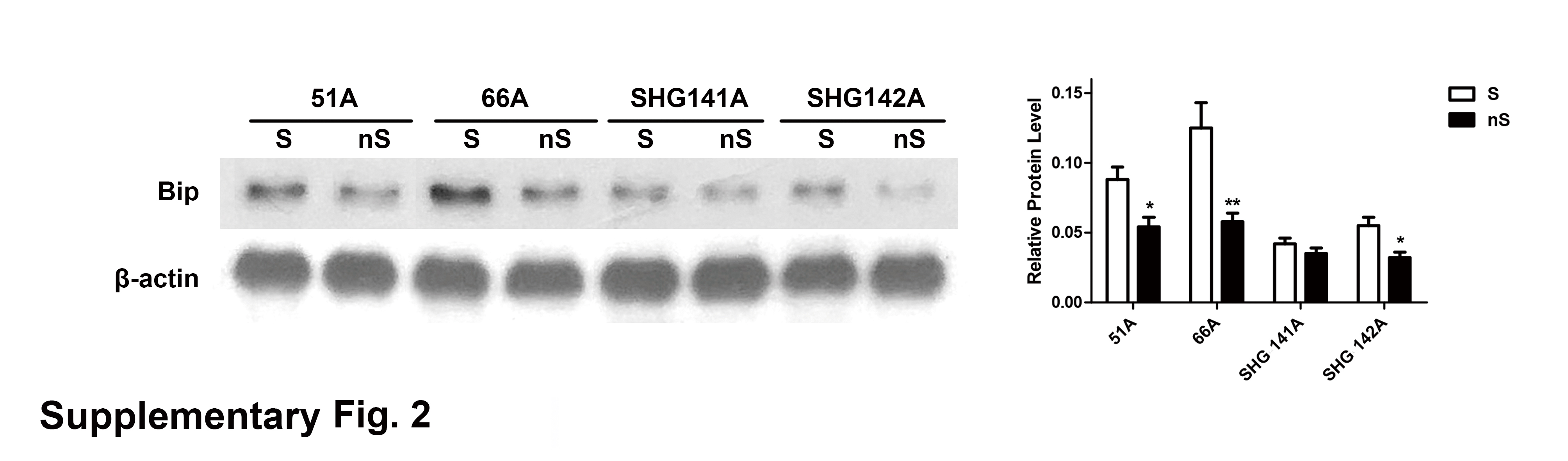

Supplement: Supplementary file 3 — Supplementary Figure 2 [file 41419_2020_3000_MOESM3_ESM.jpg]

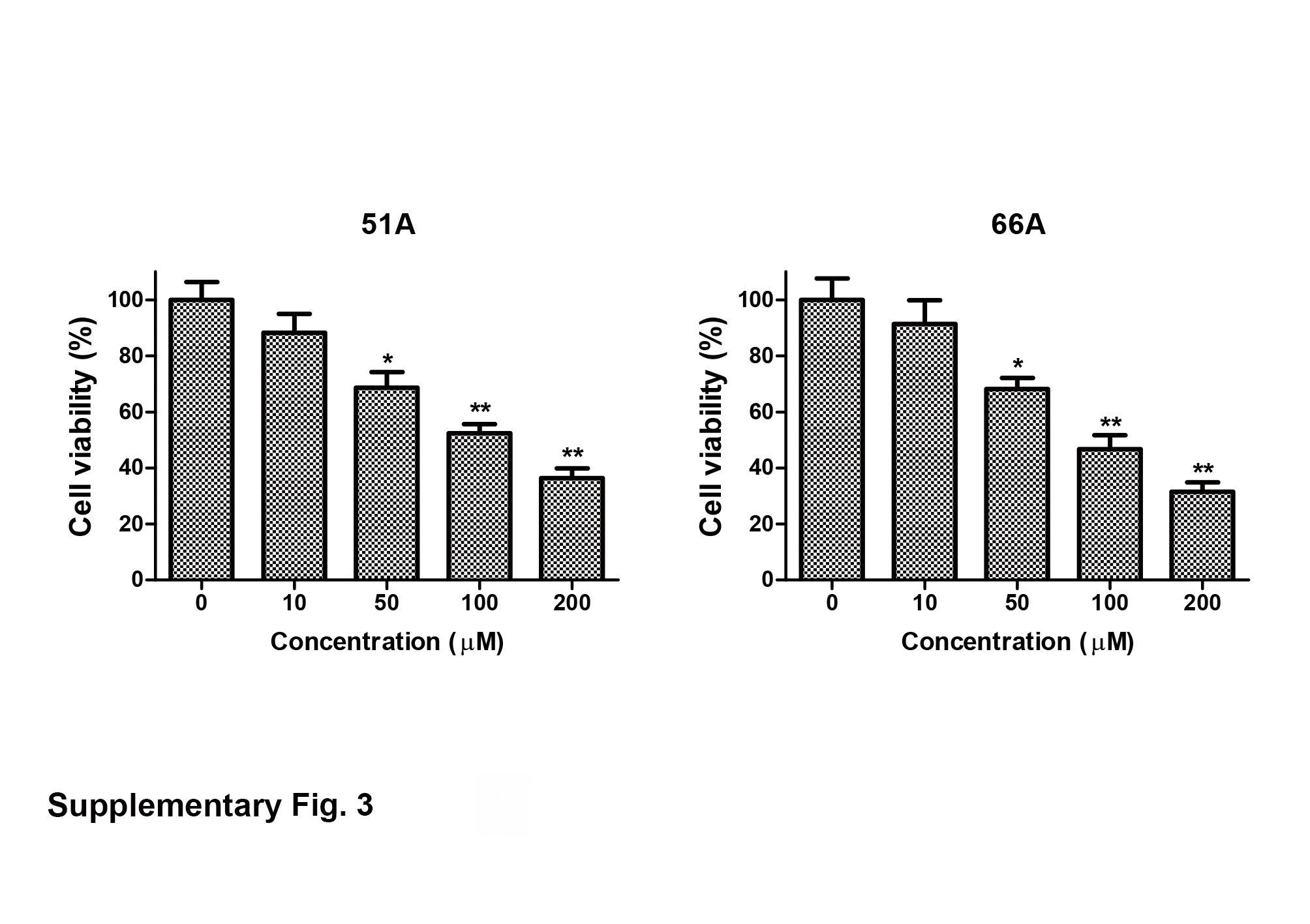

Supplement: Supplementary file 4 — Supplementary Figure 3 [file 41419_2020_3000_MOESM4_ESM.jpg]

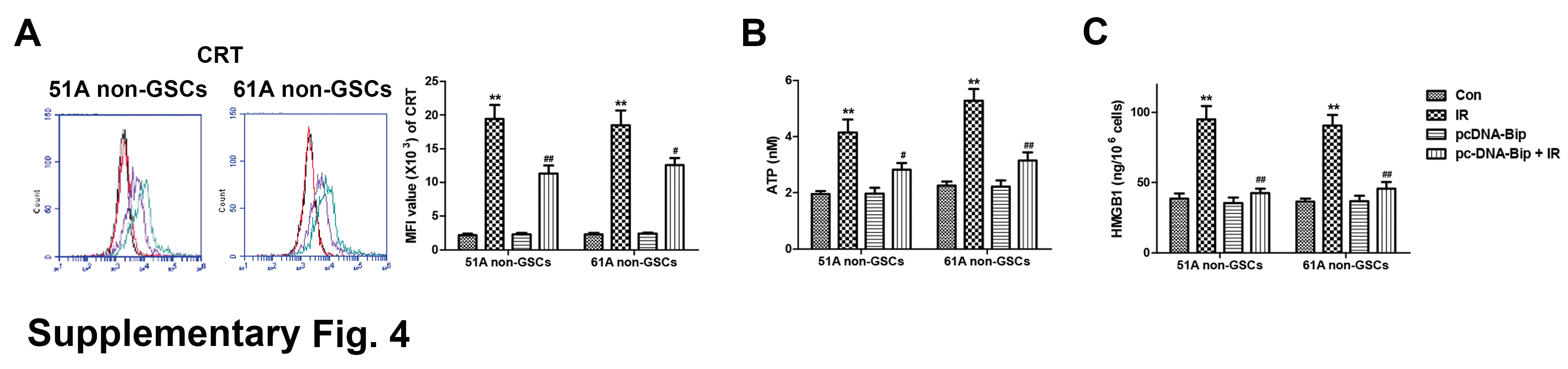

Supplement: Supplementary file 5 — Supplementary Figure 4 [file 41419_2020_3000_MOESM5_ESM.jpg]
